# The role of teacher empathy and immediacy in Chinese EFL students’ affective learning outcomes: unveiling the associations

**DOI:** 10.3389/fpsyg.2023.1294891

**Published:** 2023-11-09

**Authors:** Nana Wang, Jiafang Kang

**Affiliations:** ^1^Higher Education Research Institute, Shandong Technology and Business University, Yantai, China; ^2^School of Educational Science, Ludong University Higher Education Post-Doctor, Yantai, China; ^3^Department of Aeronautical Communication, Naval Aviation University, Yantai, China

**Keywords:** EFL learner, EFL teacher, learners’ affective learning outcomes, teacher empathy, teacher immediacy

## Abstract

**Introduction:**

The positive influences of teacher-student interpersonal communication skills on second language (L2) students have been considerably endorsed in the literature. However, the contribution of teacher empathy and immediacy behaviors, as realizations of such skills, to students’ affective learning outcomes is unaddressed in L2 research.

**Methods:**

To fill this gap, three scales were distributed among a sample of 350 Chinese EFL students to see if teachers’ empathy and immediacy correlate with and predict students’ affective learning outcomes.

**Results:**

The results of correlation analysis revealed strong and positive relationships among teacher empathy, teacher immediacy, and learners’ affective learning outcomes. Moreover, the results of multiple regression indicated that Chinese EFL teachers’ empathy and immediacy could predict about 65 and 60% of changes in the learners’ affective learning outcomes, respectively.

**Discussion:**

Implications of the study for EFL teachers’ interpersonal communication skills development and emotional literacy are discussed. Future research trends are also presented at the end of the article.

## 1. Introduction

There is a bulk of research suggesting that second language (L2) education is basically an emotional job (Mercer, 2020; Derakhshan et al., 2023a,b). Teachers’ competencies in this field are no longer limited to their pedagogical-linguistic expertise in designing classroom tasks, methods, and tests (Seligman, 2011; Hu and Wang, 2023; Wang and Derakhshan, 2023). Instead, their emotional literacy and social–emotional skills play a major role (Liu, 2016; Fu and Wang, 2022). Students feel more connected and engaged in classrooms, where their teachers are cognizant of learner-emotions (Wang et al., 2021). Teachers’ interpersonal communication behaviors and positive emotions are crucial for L2 students’ academic success, attainment, and engagement (Xie and Derakhshan, 2021; Wang Y. et al., 2022). Since teachers are the most prominent agents and players of education, their emotions and affective awareness influence instructional effectiveness and interpersonal bonds in the class (Derakhshan, 2022). Such an emotion-based education calls for teachers’ inter-emotionality with students (Wang and Jiang, 2022; Derakhshan et al., 2023a,b). It develops several positive emotions and factors in L2 learning (Shao et al., 2020). Teacher empathy is one of the outputs of this trend, which highlights one’s ability to understand and empathize with others’ perspectives and feelings (Wambsganss et al., 2022). It is a realization of emotional intelligence (EI) and social cognition that positively influences learners’ academic performance, engagement, and learning attitudes (Kianinezhad, 2023; Zhi et al., 2023). According to Stebletsova and Torubarova (2017), empathy pertains to an individual’s emotional magnanimity and care for others. Previous research shows that teacher empathy is multi-dimensional and influenced by numerous personal, affective, cognitive, contextual, and cultural factors (Stojiljković et al., 2012; Pidbutska et al., 2021; Wink et al., 2021). It can be cultivated in L2 classrooms to improve learning and regulate positive and negative emotions perceived by learners (Aldrup et al., 2022; Wang J. et al., 2022; Pan et al., 2023).

Another consequence of teacher empathy is the establishment of a strong and caring teacher-student rapport in the class (Lam et al., 2011; Wang Y. et al., 2022; Hu and Wang, 2023; Zhi and Wang, 2023). According to Xie and Derakhshan (2021), rapport is meaningfully built in an environment in which L2 teachers have interpersonal communications skills. One of those skills is teacher immediacy behaviors, which refer to the use of several verbal and non-verbal cues to create proximity and closeness with students in the class (Dickinson, 2017). It has been reported that teachers’ immediacy positively contributes to L2 students’ self-regulation, motivation, engagement, and willingness to communicate (Derakhshan, 2021; Hiver et al., 2021; Wang and Derakhshan, 2023).

Teacher immediacy also generates a sense of liking in the class and reduces the psychological distance between teachers and students (Dickinson, 2017; Dai and Wang, 2023). When teachers show empathy to their students and frequently use immediacy behaviors, the students experience fewer negative emotions (Qin, 2022; Wang, 2023). Despite these studies, the collective impact of teacher empathy and immediacy on English as a foreign language (EFL) students’ affective learning outcomes (ALOs) has remained unaddressed. The concept of ALO concerns students’ perceptions, beliefs, and attitudes toward their education (McCroskey et al., 1985). It refers to students’ outlook and emotional state perceived for the course and the instructor (Wang, 2021; Hu and Wang, 2023). Depending on teachers’ emotioncy, ALOs may be affected by classroom rapport and immediacy behaviors (Yong, 2019). However, scholarship in this regard is scant and extrapolations based on a couple of studies are unwarranted. The current literature on the three variables (empathy, immediacy, and ALOs) has focused on their separate influences on other teacher-and-learner-related constructs proposed by positive psychology. Nevertheless, their interplay in a single empirical study is absent in this area of research. To fill this gap, the present study intended to examine the predicting role of teachers’ empathy and immediacy in EFL students’ ALOs. Additionally, it aimed to test their correlations. Unmasking the associations among these constructs is significant for L2 education since L2 educators realize the idea that positive emotions are contagious and transmittable from teachers to learners. In addition, the study is prominent in that it foregrounds the linkage and exponential impact of teacher interpersonal communication abilities on learners’ emotions in L2 settings. The social, emotional, and relational aspects of L2 teaching are also highlighted in light of this study.

## 2. Literature review

### 2.1. The concept of empathy: definitions and components

The notion of empathy has to do with a person’s capacity to understand and connect with others’ emotions (Eisenberg et al., 2014). It is the manifestation of one’s opinions and feelings that end in a sense of wellbeing (Weisz and Cikara, 2021). The construct of empathy is an interpersonal behavior that indicates effort to recognize and respond to others’ thinking and feeling (Amicucci et al., 2021). According to Baron-Cohen and Wheelwright (2004), empathic people can efficiently predict others’ behaviors. Empathy has multiple dimensions such as ethical, cognitive, affective, and interactional dimensions (Mercer and Reynolds, 2002). However, the dichotomy of cognitive and affective empathy has been most popular. The former refers to one’s understanding of others’ emotional engagement, while the latter concerns sharing emotions with others (Reniers et al., 2009). In this study, empathy includes both affective and cognitive constituents. Simply, empathy is an aptitude rather than attitude for social–emotional interactions (Eisenberg et al., 2014). It is an essential skill for both teachers and learners, yet its cultivation largely hinges upon teachers.

### 2.2. Teacher empathy

Teacher empathy is described as the ability to detect, understand, and engage with students’ concerns, perspectives, and emotions through their eyes (Tettegah and Anderson, 2007). It is a significant factor in education since it provides support and interaction (Stojiljković et al., 2012). The construct develops teachers’ and learners’ mutual understanding and communal awareness (Arghode et al., 2013). Teacher empathy is best seen as an aptitude to communicate with students to provide a friendly learning environment for them to thrive (Tettegah and Anderson, 2007; Wink et al., 2021; Hu and Wang, 2023). Research shows that this factor is dynamic and changeable in relation to several factors including demographics and psycho-affective variables (Pidbutska et al., 2021). Empathetic teachers know their pupils’ positive and negative emotions and take appropriate approaches to manage such feelings (Weisz et al., 2020). In teaching, the concept of empathy is a part of teachers’ social–emotional competence that fosters classroom management (Aldrup et al., 2022). By showing empathy to their students, teachers can directly develop their psycho-social competencies such as confidence, self-concept, interpersonal interactions, and learning motivation (Wagner et al., 2016).

These contributions of teacher empathy to education are supported by the affective filter hypothesis (Krashen, 1982) and social constructivist theories (Berger and Luckmann, 1996). According to the affective filter hypothesis, students learn best when the affective filter is low. As one of the factors significant in this theory, teacher empathy establishes a low-anxiety context for learning. Therefore, it develops learning in several domains. From the social constructivism angle, teacher empathy highlights joint understandings and interactions among community members that lead to social reality emergence (Berger and Luckmann, 1996). Since education is a social entity, empathy is definitely a major player in its success. However, numerous issues and factors must be taken into account to gain academic success via empathy. One such factor is teacher immediacy, which is described below.

### 2.3. The concept of (teacher) immediacy

As one of the most important interpersonal behaviors in positive psychology, immediacy concerns the degree of psychophysical proximity among people (Mehrabian, 1969). It is the use of various signals and channels (verbal, and nonverbal) to get closer to students in the classroom (Derakhshan, 2021). Teachers’ verbal immediacy is done through humor, discussion, rapport, and praise (Gorham, 1988; Hu and Wang, 2023). They are vocal and expressive messages sent by teachers in an open classroom interaction (Ballester, 2013). Yet, non-verbal immediacy refers to body language, posture, and expressions that show closeness (Richmond et al., 1987). They are behaviors and cues related to the utilization of different strategies in relation to time (chronemics), paralinguistic features (vocalics), distance (proxemics), eye contact (oculesics), touch (haptics), body movement (kinesics), and classroom arrangements (Stilwell, 2018; Derakhshan et al., 2023b). Regardless of their typology, teacher immediacy behaviors play a critical role in academic performance and engagement of students (Delos Reyes and Torio, 2020). Since the goals of teachers and students interact in many areas, showing positive interpersonal behaviors like immediacy is vital. This joint influence is posited by the rhetorical/relational goal theory (RRGT), which regards immediacy as a determinant factor in meeting students’ academic goals (Frymier et al., 2019). Another theory behind teacher immediacy is the attachment theory (AT), which stresses out the prominence of relational patterns and emotional links among individuals (Bowlby, 1969). In academia, this sense of attachment between the teacher and students develops engagement and motivation to learn (Wang and Guan, 2020). However, the functionality and realization of immediacy depend on culture, context, and many other psycho-affective constructs (Kelly and Gaytan, 2020). Hence, it is likely that teachers’ immediacy behaviors interact with their own sense of empathy and learners’ ALOs, as described in the coming section.

### 2.4. Students’ affective learning outcomes

It is believed that teacher-student relationships and interpersonal communication qualities influence learners’ ALOs (Yong, 2019). The concept of ALOs denotes students’ perceptions and attitudes toward learning (Witt and Wheeless, 2001). It reflects learners’ overall view of teaching, learning, the course, classroom materials, and the teacher (Pogue and AhYun, 2006). Operationally, ALOs points to students’ social, emotional, and attitudinal aspects of learning that exert impact on their learning experience. As put by Bekiari (2012), teachers’ proximity and interpersonal communication with students can radically influence their emotionality and learning. Such affective outcomes are significant in educational contexts as they produce and re-produce many other positive emotions (Pekrun et al., 2011; Pan et al., 2023). If ALOs are positive and optimal, students are more likely to succeed in academic domains (Goodboy et al., 2015). Previous research pinpoints the malleable and sensitive nature of ALOs in that they are affected by learner-related and teacher-related factors (Wang and Guan, 2020). It is also reported that teacher-learner communication and rapport are the antecedents of ALOs. Therefore, it is logical to extrapolate that the presence of teacher empathy and immediacy can predict EFL students’ ALOs. However, this line of research requires further evidence.

### 2.5. Previous studies

Educational research shows that teachers’ interpersonal communication behaviors considerably affect various aspects of L2 students’ learning process (Xie and Derakhshan, 2021; Derakhshan, 2022). As an important interpersonal factor, teacher empathy has been the focus of different L2 studies in the past decades. For example, Wang and Guan (2020) argued that teacher empathy is significantly correlated with learners’ sense psychological wellbeing in China. Likewise, Wink et al. (2021) maintained that empathetic teachers are more likely to form positive mindsets about students’ performance and behaviors. Teacher empathy has also been found correlated with teachers’ professional identity (Zhu et al., 2019), satisfaction (Hassanpour Souderjani et al., 2021), and emotional intelligence (Salem and Tabatabaei, 2015). As for learners, Kianinezhad (2023) ran a study in Iran and discovered a positive relationship between teacher empathy and EFL students’ classroom engagement. In the same context, Karimian (2022) conducted an experimental study on 60 EFL students and found a positive association between teacher empathy and students’ empathy and language achievement, as shown in the experimental group. In a conceptual study, in China, Zhang (2022) provided a comprehensive guide on how teacher empathy can contribute to L2 learners’ engagement. The antecedents of teacher empathy have also been examined in the literature including demographic factors, fantasy, autonomy, identity, compassion, and apprehension (Csaszar et al., 2018; Zhu et al., 2019; Zohoorian and Faravani, 2021).

Although the interaction of teacher empathy and teacher-student interpersonal communication has been endorsed in the literature (Aldrup et al., 2022), the possible link between empathy and immediacy among EFL teachers has been ignored. Teacher immediacy, itself, has gained a surge of attention from L2 researchers working on interpersonal communication (Kelly and Gaytan, 2020; Derakhshan, 2021). In a recent study, Zheng (2021) posited that teacher immediacy could enhance EFL learners’ clarity, credibility, motivation, and engagement in English classes. Moreover, Hiver et al. (2021) found teachers’ immediacy behaviors beneficial for learners’ motivation and self-regulation. The association between teacher immediacy and many other variables such as socio-affective development, adaptability, communication skills, and negative emotion regulation has been reported in the literature (Bolkan et al., 2017; Pishghadam et al., 2019; Zheng, 2021; Qin, 2022). While these studies have been insightful, they have overlooked the possible relationship between teacher empathy and immediacy. Additionally, there is a limited scholarship concerning the predicting power of these two variables in EFL students’ affective outcomes. The construct of ALOs, compared to the other two constructs in this study, has received a slight attention in L2 research. In a seminal work, Sun and Shi (2022) examined the correlation among classroom rapport, teacher support, and ALOs among 497 Chinese EFL students. The results of their study indicated that teacher-student rapport and teacher support positively correlated with students’ ALOs. The literature on this variable is dearth, especially in L2 education. To cast some light on the relationship between two teacher-related variables (empathy, immediacy) and EFL students’ ALOs, this study used a quantitative design in the context of China. So far, the literature has mostly concentrated on the contribution of teacher empathy and immediacy to learners’ communication skills, while their facilitative role in affective outcome is widely ignored. Such an interaction is significant in that the transmittable effect of teachers’ psycho-emotional factors and interpersonal communication skills on EFL students’ affect and learning would be brought to the fore in L2 research. Specifically, the present research sought to answer the ensuing research questions:

1. Are there any significant relationships between teacher empathy, teacher immediacy, and students’ affective learning outcomes?
2. Do EFL teachers’ empathy and immediacy significantly predict their students’ affective learning outcomes?

## 3. Methods

### 3.1. Research design

This study used a correlation research design as a common design in descriptive quantitative studies. The purpose of choosing this type of design was unraveling the association among the three constructs of concern and depicting the strength of such an association. Moreover, this design fitted well with the objectives of this study, which were determining the relationships, changes, and predictive powers of the variables.

### 3.2. Participants and context

A total of 350 Chinese EFL students took part in this study. They came from different provinces including Henan, Guangxi, Shaanxi, and Liaoning. The participants were studying at colleges and high schools in these provinces. The number of females (55.14%) surveyed was slightly higher than the number of males (44.86%). Participants aged between 35 and 39 years old. Of the sample, 41.43% were undergraduates, 21.14% were high school students, 19.71% were postgraduates, and 17.71% were doctoral students. They were selected non-randomly through a convenience sampling technique. Their majors included applied linguistics (35%), English literature (30%), translation studies (28%), and linguistics (7%). The participants expressed their willingness and consent before attending the survey. This ethical concern was met by sending emails and invitation messages through social media to the target participants.

### 3.3. Instruments

#### 3.3.1. Teacher empathy scale

To assess teacher empathy, Barrett-Lennard’s (2015) valid scale was used that encompassed 40 items. The respondents had to rate their answers in a scale with positive and negative sides. In the positive side, each item was scored from +1 (I feel that it is probably true, or more true than untrue) to +3 (I strongly feel that it is true), while the negative side ranged from-1 (I feel that it is probably untrue, or more untrue than true) to-3 (I strongly feel that it is not true). Three dimensions of cognitive empathy, negative affective empathy, and positive affective empathy constituted the scale. The last version of the questionnaire was piloted with 50 participants of the same population. The reliability index of Cronbach’s alpha was estimated to be 0.93, which is a high index. “I feel s/he is pleased to see me” is a sample item from the scale.

#### 3.3.2. Teacher immediacy scale

To measure teacher immediacy behaviors, a modified valid scale developed by Gorham (1988) and Richmond et al. (1987) was used in this study. It included 33 items based on a 5-point Likert scale ranging from 1 (Never) to 5 (Very often). The scale had two dimensions of verbal immediacy (20 items) and nonverbal immediacy (13 items). “My teacher asks questions or encourages students to talk” is a sample item in the scale. The last version of the questionnaire was piloted with 50 participants of the same population. The reliability index of Cronbach’s alpha was estimated to be 0.85, which is acceptable.

#### 3.3.3. Students’ affective learning outcomes scale

Regarding this construct, McCroskey et al.’s (1985) valid scale was used. It comprised 16 items under 5 dimensions of attitude toward the course content (5 items), attitudes toward behaviors recommended in the course (4 items), attitude about the teacher (4 items), likelihood of taking another course with this teacher (2 items), and actual engagement in the behaviors recommended in the course (2 items). A 7-point Likert scale was used in this tool, but with different labels representing the options. The last version of the questionnaire was piloted with 50 participants of the same population. The reliability index of Cronbach alpha was estimated to be 0.89, which is acceptable. As an example, “my attitude about the content in this course is…” is one of the items in this scale.

### 3.4. Data collection procedure

In this study, the researchers distributed an electronic version of the three scales described above among a sample of 400 EFL students of whom 350 respondents delivered the data completely. The direct link of the questionnaires was shared with the participants in China via email and ‘WeChat’. To make the respondents feel at ease, both English and Chinese languages were allowed to answer the items. The participants were informed about the objectives of the study. They also gave their consent to cooperate formally. Some electronic books related to English learning and research were given to the respondents as an appreciation of their devoted time and energy. The data were collected in 2 days and was completed on March 9. Only questionnaires were used to collect data, and no other research methods were used. The survey was conducted in full compliance with the Research Ethics guidelines, and participants were aware that the information provided would be kept confidential and used only for research purposes. The researchers had no conflict of interest with any of the participants. After collecting the questionnaires’ data, the researchers checked them carefully to ensure their accuracy and authenticity. Afterwards, the data were organized and entered into an Excel file to be fed into SPSS and Amos software for the final analysis.

### 3.5. Data analysis

To analyze the data and answer the formulated questions, the researchers used SPSS software (v. 27) and AMOS (v. 24). Additionally, structural equation modeling (SEM) was employed to draw a possible model of the association among teacher empathy, immediacy, and ALOs. Descriptive statistics, reliability analysis, correlation, and multiple linear regression were also used to examine the collected data.

## 4. Results

The non-significant values of Kolmogorov–Smirnov test show that the assumption of normality was not violated. Therefore, the researchers used parametric test to analyze the obtained data (Table 1).

**Table 1:** Test of normality.

	Kolmogorov–Smirnov^a^		
	Statistic	DF	Sig.
Student affective learning	64.6	350	0.625
Teacher empathy	140.2	350	0.536
Teacher immediacy	95.7	350	0.741

^a^Lilliefors significance correction.

**Figure 1:**
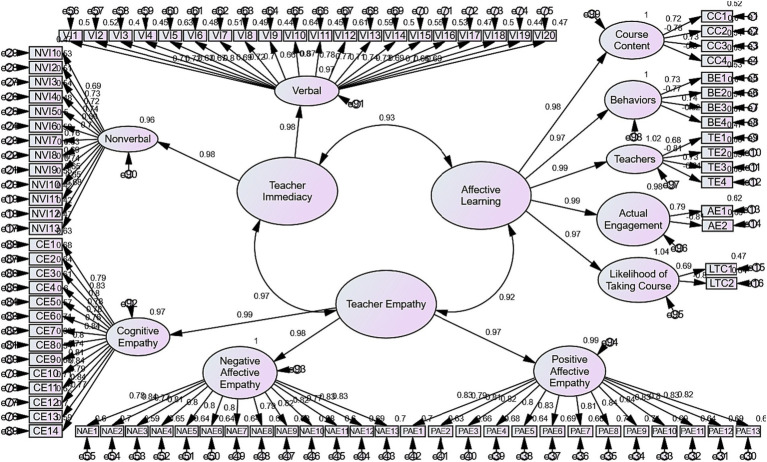
The final modified CFA model with standardized estimates.

To answer the first research question and to acknowledge the convergent validity of the relationship between ALOs, teacher empathy, and teacher immediacy, CFA was run. The initial model showed good fit to the data (see Figure 1). Goodness-of-fit indices can be seen in Table 2.

**Table 2:** Evaluation of the CFA goodness of fit.

		Threshold			
Criteria		Terrible	Acceptable	Excellent	Evaluation
CMIN	11473.660				
DF	3,814				
CMIN/DF	3.008	>5	>3	>1	Acceptable
RMSEA	0.076	>0.08	<0.08	<0.06	Acceptable
GFI	0.967	<0.9	>0.9	>0.95	Acceptable
CFI	0.966	<0.9	>0.9	>0.95	Acceptable
PNFI	0.669	<0.5	>0.5		Acceptable
TLI	0.960	>0.9	>0.9	>0.95	Acceptable

In Table 2, the result indicated that five determiners are ratio of CMIN-DF, goodness-of-fit index (GFI), comparative fit index (CFI), Parsimonious Normed Fit Index (PNFI), Tucker–Lewis Index (TLI), and root mean square error of approximation (RMSEA). The model fit indices are all within specifications. Therefore, CMIN/DF is 3.008 (spec. ≤3.0), GFI = 0.967 (spec. >0.9), CFI = 0.966 (spec. >0.9), PNFI = 0.669 (spec. >0.5), TLI = 0.960 (spec. >0.9), and RMSEA = 0.076 (spec. <0.080).

The results of Table 3 show that composite reliabilities of the factors are acceptable (CR > 0.70). In other words, the model has achieved composite reliability. The values also demonstrate that the convergent validity of the factors reach to an acceptable value (AVE > 0.50) or the model has achieved convergent validity. Another requirement of convergent validity is factor loading more than 0.50. The results of factor loading are presented in Table 4. In addition, the results indicate that the model has achieved discriminant validity (the square root of AVE > inter-construct correlations).

**Table 3:** Composite reliability and discriminant validity of the factors.

	CR	AVE	MSV	MaxR(H)	Student affective learning	Teacher empathy	Teacher immediacy
Student affective learning	0.896	0.835	0.251	0.922	**0.934**		
Teacher empathy	0.932	0.827	0.146	0.965	0.146	**0.918**	
Teacher immediacy	0.854	0.768	0.167	0.924	0.375	0.189	**0.867**

**Table 4:** Factor loading of the initial CFA model.

			Estimate	S.E.	C.R.	*p*
Affective learning	<-->	Teacher immediacy	1.050	0.117	−9.011	0.000
Teacher empathy	<-->	Affective learning	1.533	0.159	9.615	0.000
Teacher empathy	<-->	Teacher immediacy	0.935	0.095	−9.811	0.000
Verbal	<---	Teacher immediacy	1.000			
Nonverbal	<---	Teacher immediacy	0.969	0.078	12.386	0.000
Cognitive empathy	<---	Teacher empathy	0.965	0.051	18.920	0.000
Negative affective empathy	<---	Teacher empathy	1.004	0.052	19.157	0.000
Positive affective empathy	<---	Teacher empathy	1.000			
Likelihood of taking course	<---	Affective learning	1.000			
Actual engagement	<---	Affective learning	1.105	0.078	14.196	0.000
Teacher	<---	Affective learning	0.930	0.074	12.535	0.000
Behavior	<---	Affective learning	1.023	0.077	13.208	0.000
Course content	<---	Affective learning	1.029	0.078	13.154	0.000
CC1	<---	Course content	1.000			
CC2	<---	Course content	−0.895	0.061	−14.698	0.000
CC3	<---	Course content	0.999	0.072	13.844	0.000
CC4	<---	Course content	−0.899	0.059	−15.238	0.000
BE1	<---	Behavior	1.000			
BE2	<---	Behavior	−0.831	0.057	−14.683	0.000
BE3	<---	Behavior	1.029	0.074	13.941	0.000
BE4	<---	Behavior	−0.962	0.062	−15.629	0.000
TE1	<---	Teacher	1.000			
TE2	<---	Teacher	−1.094	0.076	−14.334	0.000
TE3	<---	Teacher	1.118	0.086	12.952	0.000
TE4	<---	Teacher	−1.078	0.075	−14.365	0.000
AE1	<---	Actual engagement	1.000			
AE2	<---	Actual engagement	−0.837	0.048	−17.282	0.000
LTC1	<---	Likelihood of taking course	1.000			
LTC2	<---	Likelihood of taking course	−0.991	0.068	−14.469	0.000
NVI13	<---	Nonverbal	1.000			
NVI12	<---	Nonverbal	0.928	0.081	11.497	0.000
NVI11	<---	Nonverbal	0.894	0.077	11.565	0.000
NVI10	<---	Nonverbal	1.159	0.089	13.090	0.000
NVI9	<---	Nonverbal	0.990	0.081	12.190	0.000
NVI8	<---	Nonverbal	0.926	0.082	11.261	0.000
NVI7	<---	Nonverbal	1.147	0.085	13.471	0.000
NVI6	<---	Nonverbal	1.127	0.090	12.494	0.000
NVI5	<---	Nonverbal	0.990	0.080	12.314	0.000
NVI4	<---	Nonverbal	1.098	0.085	12.991	0.000
NVI3	<---	Nonverbal	1.164	0.092	12.680	0.000
NVI2	<---	Nonverbal	1.102	0.085	12.916	0.000
NVI1	<---	Nonverbal	0.934	0.077	12.183	0.000
PAE13	<---	Positive affective empathy	1.000			
PAE12	<---	Positive affective empathy	1.023	0.054	19.100	0.000
PAE11	<---	Positive affective empathy	1.003	0.056	18.020	0.000
PAE10	<---	Positive affective empathy	0.967	0.051	19.065	0.000
PAE9	<---	Positive affective empathy	1.073	0.055	19.521	0.000
PAE8	<---	Positive affective empathy	1.092	0.056	19.567	0.000
PAE7	<---	Positive affective empathy	0.993	0.054	18.460	0.000
PAE6	<---	Positive affective empathy	1.030	0.054	19.013	0.000
PAE5	<---	Positive affective empathy	0.929	0.051	18.070	0.000
PAE4	<---	Positive affective empathy	1.053	0.056	18.844	0.000
PAE3	<---	Positive affective empathy	0.979	0.053	18.542	0.000
PAE2	<---	Positive affective empathy	0.878	0.049	17.744	0.000
PAE1	<---	Positive affective empathy	1.047	0.054	19.244	0.000
NAE13	<---	Negative affective empathy	1.000			
NAE12	<---	Negative affective empathy	1.026	0.052	19.601	0.000
NAE11	<---	Negative affective empathy	1.000	0.057	17.656	0.000
NAE10	<---	Negative affective empathy	1.017	0.052	19.443	0.000
NAE9	<---	Negative affective empathy	1.011	0.052	19.369	0.000
NAE8	<---	Negative affective empathy	1.048	0.058	18.061	0.000
NAE7	<---	Negative affective empathy	0.951	0.051	18.571	0.000
NAE6	<---	Negative affective empathy	0.880	0.047	18.627	0.000
NAE5	<---	Negative affective empathy	1.028	0.055	18.647	0.000
NAE4	<---	Negative affective empathy	0.930	0.049	18.893	0.000
NAE3	<---	Negative affective empathy	0.890	0.051	17.382	0.000
NAE2	<---	Negative affective empathy	1.029	0.052	19.909	0.000
NAE1	<---	Negative affective empathy	0.992	0.056	17.716	0.000
VI1	<---	Verbal	1.000			
VI2	<---	Verbal	1.076	0.080	13.393	0.000
VI3	<---	Verbal	0.860	0.074	11.654	0.000
VI4	<---	Verbal	0.943	0.076	12.435	0.000
VI5	<---	Verbal	1.177	0.080	14.731	0.000
VI6	<---	Verbal	0.955	0.075	12.770	0.000
VI7	<---	Verbal	1.048	0.079	13.259	0.000
VI8	<---	Verbal	0.998	0.077	12.904	0.000
VI9	<---	Verbal	0.823	0.067	12.224	0.000
VI10	<---	Verbal	1.196	0.081	14.803	0.000
VI11	<---	Verbal	0.926	0.074	12.437	0.000
VI12	<---	Verbal	1.110	0.077	14.443	0.000
VI13	<---	Verbal	1.138	0.080	14.192	0.000
VI14	<---	Verbal	1.005	0.077	13.121	0.000
VI15	<---	Verbal	1.009	0.073	13.757	0.000
VI16	<---	Verbal	1.077	0.080	13.508	0.000
VI17	<---	Verbal	0.911	0.072	12.729	0.000
VI18	<---	Verbal	0.889	0.068	13.034	0.000
VI19	<---	Verbal	1.011	0.082	12.276	0.000
VI20	<---	Verbal	1.106	0.087	12.695	0.000
CE13	<---	Cognitive empathy	1.000			
CE12	<---	Cognitive empathy	0.917	0.050	18.211	0.000
CE11	<---	Cognitive empathy	1.005	0.050	20.200	0.000
CE10	<---	Cognitive empathy	1.023	0.054	18.947	0.000
CE9	<---	Cognitive empathy	0.935	0.057	16.451	0.000
CE8	<---	Cognitive empathy	0.992	0.054	18.417	0.000
CE7	<---	Cognitive empathy	1.026	0.051	20.295	0.000
CE6	<---	Cognitive empathy	0.966	0.057	17.067	0.000
CE5	<---	Cognitive empathy	1.154	0.065	17.751	0.000
CE4	<---	Cognitive empathy	0.926	0.051	17.989	0.000
CE3	<---	Cognitive empathy	1.053	0.057	18.495	0.000
CE2	<---	Cognitive empathy	0.995	0.051	19.627	0.000
CE1	<---	Cognitive empathy	1.015	0.055	18.324	0.000
CE14	<---	Cognitive empathy	0.998	0.057	17.437	0.000

The results of Table 4 show that almost all of the values are more than 0.50. It means that the model has achieved the convergent validity.

**Figure 2:**
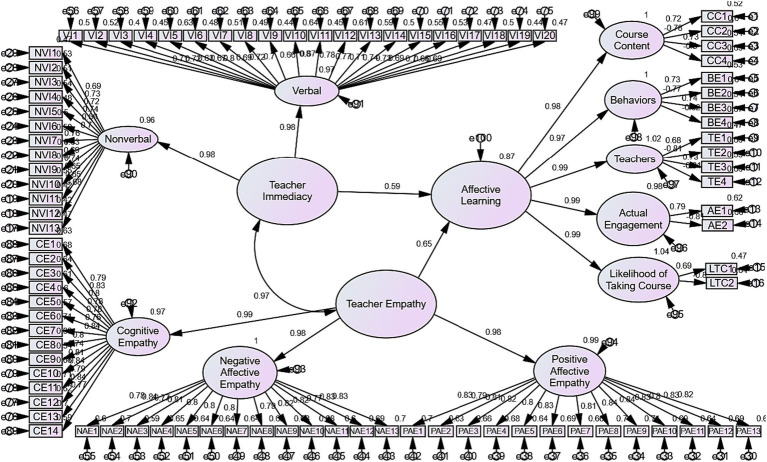
The final measurement model with standardized estimates.

**Figure 3:**
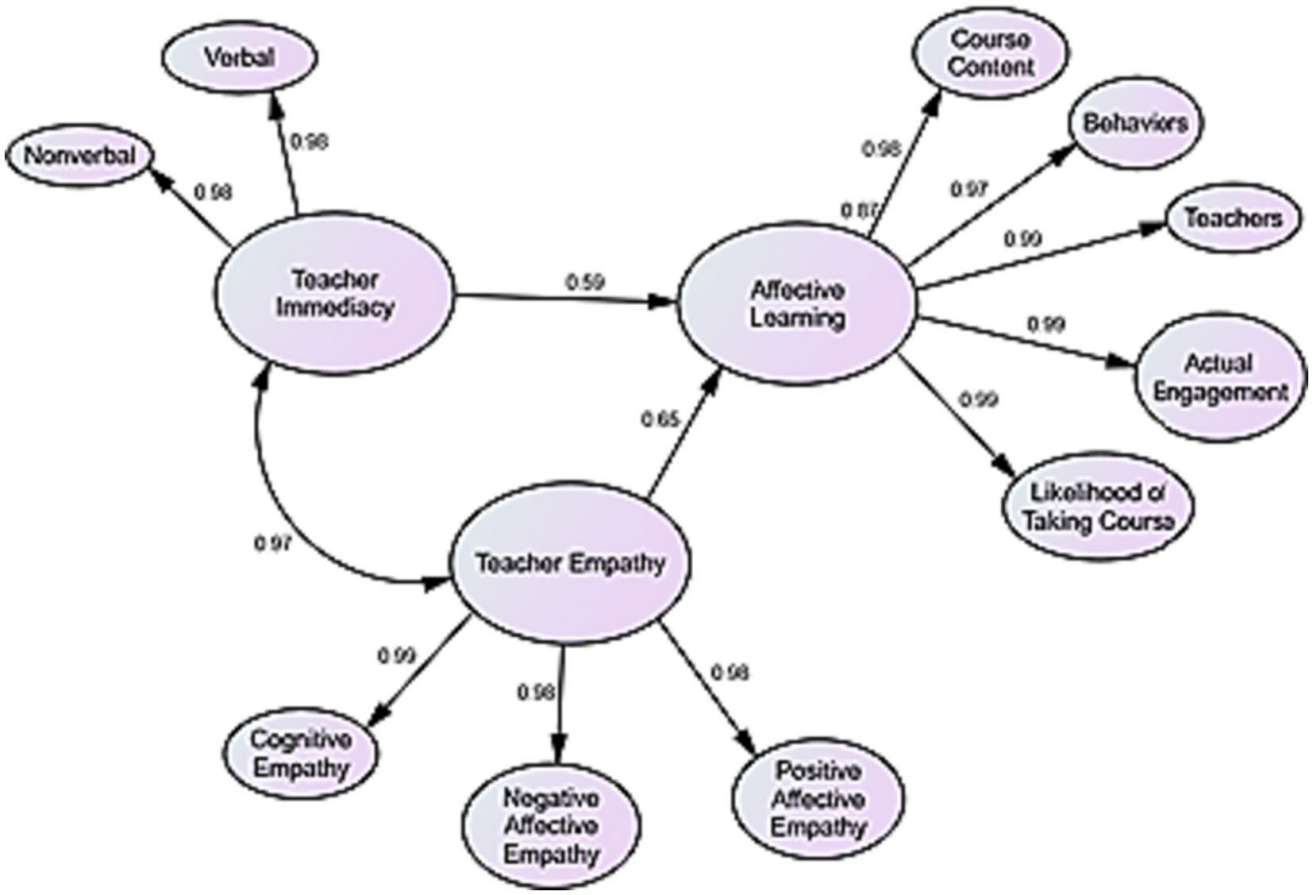
The final measurement model.

The results of Table 5 indicate that there are strong positive relationships between teacher immediacy, teacher empathy, and learners’ ALOs. To answer the research question, Linear Regression was run in SEM. The results of this analysis are presented in Table 6 and Figures 2, 3.

**Table 5:** The relationships between the variables.

			Estimate
Affective learning	<-->	Teacher immediacy	0.928
Teacher empathy	<-->	Affective learning	0.920
Teacher empathy	<-->	Teacher immediacy	0.967

**Table 6:** Results of linear regression analysis with SEM.

			Estimate	S.E.	C.R.	*p*
Affective learning	<---	Teacher immediacy	0.593	0.117	−9.011	0.000
Teacher empathy	--->	Affective learning	0.654	0.159	9.615	0.000
Teacher empathy	<-->	Teacher immediacy	0.976	0.095	−9.811	0.000
Verbal	<---	Teacher immediacy	0.984			
Nonverbal	<---	Teacher immediacy	0.996	0.078	12.386	0.000
Cognitive empathy	<---	Teacher empathy	0.993	0.051	18.920	0.000
Negative affective empathy	<---	Teacher empathy	0.992	0.052	19.157	0.000
Positive affective empathy	<---	Teacher empathy	0.987			
Likelihood of taking course	<---	Affective learning	0.993			
Actual engagement	<---	Affective learning	0.994	0.078	14.196	0.000
Teacher	<---	Affective learning	0.985	0.074	12.535	0.000
Behavior	<---	Affective learning	0.982	0.077	13.208	0.000
Course content	<---	Affective learning	0.985	0.078	13.154	0.000

The results of Table 6 represent that about 60% of changes in the learners’ ALOs can be predicted by their teacher immediacy; and about 65% of changes in the learners’ ALOs can be predicted by their teacher empathy.

## 5. Discussion

This study aimed at testing a hypothesized model of the association among Chinese EFL teachers’ empathy, immediacy, and ALOs from the perspective of students. The results showed that teacher empathy and immediacy had strong and positive relationships with students’ ALOs. This is theoretically consistent with constructivist theories, the affective filter hypothesis, RRGT, and AT, which highlight the role of emotional bonds and joint construction of reality in the classroom. In particular, the results echo social constructivism in that teacher empathy and immediacy behaviors are two important factors in social interaction and development. One cannot develop interactional skills without a knowledge of empathy and immediacy in the classroom. This expertise is then transferred to students in the class. Additionally, the presence of these variables lowers affective filters and tensions among students. Hence, their academic performance and success is more likely in a friendly context. The results align with RRGT in that teacher empathy and immediacy affect the joint relational patterns between the teacher and his/her students. Education is a joint social process, which demands interpersonal skills and emotional awareness. When these skills are present, several positive ALOs will pop up in the language learning process. Finally, the results concur with AT in that all the three constructs of concern in this study reflect relational patterns and affective connections in the classroom to academically thrive. Empirically, the results are comparable with Derakhshan (2021) and Kelly and Gaytan (2020), who argued that teachers’ interpersonal communication skills and emotional skills significantly contribute to students’ positive emotional outcomes. Likewise, the positive interaction among the variables is in line with Aldrup et al. (2022), who carried out a systematic review on the association between teacher-student interactions and students’ outcomes. They argued that high levels of interpersonal communication skills incur positive feelings in learners. A reason for this finding could be the participants’ high emotional literacy and knowledge of positive psychology in L2 education. It is also likely that the students had been cognizant of the joint, affective, and social foundation of L2 education making them being concerned about interpersonal communication skills in an emotional world. It also seems that the participants had received enough training on the psychology of English language education in China. Another explanation for the gained result could be the Chinese students’ preferences to engage in interpersonal communication encounters with their teachers as a way to express and form academic emotions in the class. In other words, they believed that interpersonal relations and communications are not detached form learner emotions and affect (Ghiasvand and Banitalebi, 2023). Last but not the least, the result can be explained by the transmitting and contagious nature of interpersonal communication skills and teacher emotions to students. In simple terms, teacher empathy and immediacy are contagious behaviors that definitely transmit to students and generate different emotions among them.

Another result in this study was that teacher empathy and teacher immediacy could separately predict about 65 and 60% of changes in the learners’ ALOs. This positive interplay resonates with Sun and Shi’s (2022) study in China, which reported a strong correlation between teachers’ interpersonal communication skills and students’ ALOs in a sample including 497 EFL students. Moreover, different studies in the literature have argued that teacher empathy and immediacy lead to other positive emotions and outcomes in L2 learners (Derakhshan, 2021; Zheng, 2021; Zhang, 2022; Kianinezhad, 2023). Again, an explanation for such predictive power might be the contagious nature of many teacher-related emotions in L2 education and their transferability to learners. Since teachers have the most influence on students’ academic life, their interpersonal communication skills and emotional literacy considerably affect students’ emotions and behaviors. The participants’ concern for others’ emotions and walking in their shoes could be the reason behind Chinese students’ perceived influence of teacher empathy and immediacy on ALOs. It is also possible that the participants’ high socio-emotional competence and emotional intelligence had made them see the interaction among the variables direct and positive. Another justification might be the idea that teacher-learner interactions are by no means emotion-free endeavors (Ghiasvand, 2022). Instead, the affectivity and emotional states of both interactants play a vital role in their interpersonal understanding and relation. It can be asserted that teacher empathy and immediacy behaviors (verbal and non-verbal) carry emotions with themselves. Like other teacher-related behaviors, these two communication skills cause positive emotions among pupils, too.

## 6. Conclusion and implications

The results of this study concluded that teachers’ interpersonal communication skills such as empathy and immediacy behaviors have penetrating influences on L2 students’ affective outcomes, as well. It can also be inferred that teachers’ emotions permeate into those of their students and they are indeed interdependent (Derakhshan et al., 2023a,b). Knowing this interconnection is pedagogically momentous for EFL teachers, who can understand the contagious and transmittable nature of their own psycho-affective factors and interpersonal skills to the students’ world. Every behavior, practice, and emotion of a teacher may leave unfathomable influences on students’ affect. Hence, EFL teachers may use this study to ponder more deeply about their interpersonal communication skills in class to produce positive outcomes among learners. This pedagogical development can be gained in training courses by teacher educators, who can use the results and design practical techniques for EFL teachers to use proper immediacy behaviors and be empathetic in the classroom so that their students’ emotions are positively stimulated. Teacher trainers can run workshops in EFL context for teachers to cultivate an emotion-based L2 education atmosphere, which can generate numerous positive results for both teachers and learners.

Although the study provides fresh ideas about the influence of teacher-student interpersonal communication skills on EFL students’ ALOs, it fails to show whether demographic, contextual, cultural, and other emotional factors played a role in such an interplay or not. It is also possible that the extracted model of associations among the construct’s changes in larger samples. These are concerns that can be examined in the future. The use of a pure quantitative design is another limitation of this study, which other research designs can complement in the future studies (Derakhshan et al., 2023a). The dynamic nature of the purported model of associations among teacher empathy, immediacy, and ALOs is also a new line for further research. The role of teachers’ emotions and interpersonal skills in their alternative assessment practices and identity development is also an interesting line of thinking in the future (Derakhshan and Ghiasvand, 2022; Estaji and Ghiasvand, 2023). Future research is invited to examine the role of other interpersonal communication skills in learners’ academic emotions. Furthermore, future researchers can study the realization of teacher empathy and immediacy in the L2 assessment world and its corresponding emotions (Banitalebi and Ghiasvand, 2023; Derakhshan et al., 2023a,b). Finally, the transition from face-to-face education to online education can be explored in light of interpersonal communication skills and students’ emotional states, as a consequence of such a shift.

## Data availability statement

The original contributions presented in the study are included in the article/supplementary material, further inquiries can be directed to the corresponding author.

## Ethics statement

The studies involving humans were approved by the Academic and Ethics Committee of Shandong Technology and Business University and Ludong University. The studies were conducted in accordance with the local legislation and institutional requirements. The participants provided their written informed consent to participate in this study.

## Author contributions

NW: Data curation, Formal analysis, Writing – original draft. JK: Methodology, Writing – review & editing.
